# Plasticity of a holobiont: desiccation induces fasting-like metabolism within the lichen microbiota

**DOI:** 10.1038/s41396-018-0286-7

**Published:** 2018-10-11

**Authors:** Tomislav Cernava, Ines Aline Aschenbrenner, Jung Soh, Christoph W. Sensen, Martin Grube, Gabriele Berg

**Affiliations:** 1 Institute of Environmental Biotechnology, Graz University of Technology, Petersgasse 12, 8010 Graz, Austria; 2 Institute of Computational Biotechnology, Graz University of Technology, Petersgasse 14, 8010 Graz, Austria; 3 BioTechMed Graz, Mozartgasse 12/II, 8010 Graz, Austria; 4 Institute of Plant Sciences, University of Graz, Holteigasse 6, 8010 Graz, Austria

**Keywords:** Microbial ecology, Microbiome

## Abstract

The role of host-associated microbiota in enduring dehydration and drought is largely unknown. We have used lichens to study this increasingly important problem because they are the organisms that are optimally adapted to reoccurring hydration/dehydration cycles, and they host a defined and persistent bacterial community. The analysis of metatranscriptomic datasets from bacterial communities of the lung lichen (*Lobaria pulmonaria* (L.) Hoffm.), sampled under representative hydration stages, revealed significant structural shifts and functional specialization to host conditions. The hydrated samples showed upregulated transcription of transport systems, tRNA modification and various porins (Omp2b by *Rhizobiales*), whereas the desiccated samples showed different functions related to stress adaption prominently. Carbohydrate metabolism was activated under both conditions. Under dry conditions, upregulation of a specialized ketone metabolism indicated a switch to lipid-based nutrition. Several bacterial lineages were involved in a functional transition that was reminiscent of a ‘fasting metaorganism’. Similar functional adaptions were assigned to taxonomically unrelated groups, indicating hydration-related specialization of the microbiota. We were able to show that host-associated bacterial communities are well adapted to dehydration by stress protection and changes of the metabolism. Moreover, our results indicate an intense interplay in holobiont functioning under drought stress.

## Introduction

Coadaptation has resulted in intimate relationships forming between microbes and their hosts that create specific and stable microbiomes [1–3]. Therefore, all eukaryotic organisms can be considered to be metaorganisms, or holobionts, i.e. assemblages including different microbial species that form ecological units with their hosts. In this perspective, the microbiome fulfils functions for maintaining the integrity of the entire organismal system [4]. Many organisms have actually ‘outsourced’ some essential functions, including aspects contributing to their own development, to symbiotic organisms living with them [5]. Mendes and Raaijmakers identified cross-kingdom similarities in microbiome functions, which include nutrient acquisition, production of vitamins, amino acids and hormones, immune system modulation, and protection against biotic and abiotic stress [6]. However, detailed knowledge related to protection strategies that are activated by host-associated microbiota in response to abiotic stresses is limited, as well as the implications for the hosts. Exploitation of microbiota-mediated protection mechanisms will likely become more important in the future, because weather conditions, including droughts and extreme rainfall events, are increasing and expected to alternate quickly [7]. It was recently shown that drought has a substantial impact on the development of the plant microbiome in sorghum and that bacterial functioning is adapted under this condition [8]. According to experimental evidence, bacterial inoculants can protect plants against drought stress [9, 10]; however, the underlying mechanisms and the functions at the community level need to be characterized further, especially with respect to functional responses of a host-associated microbial community to this prevalent abiotic stress.

Lichens are ideal models to study the response of the microbiome to desiccation, because it is an integral part of their life style and survival strategy [11, 12], which even allows them to survive under space conditions when dried out [13]. Once in the dehydrated stage, rapid hydration, which can occur during a rainfall or high humidity in the morning hours, will cause the formation of oxygen radicals in the algal photosystems [14]. Therefore, lichens have additionally evolved effective protective mechanisms against oxidative stress and for the resumption of metabolism following rehydration [15, 16], which allow them to exist in a wide range of habitats, including places where higher plants cannot survive [11, 13]. Previous studies of mechanisms conferring this remarkable desiccation tolerance have focused on the primary partners contributing to the lichen thallus in the context of different hydration levels [16–18]. Many unsolved questions remain as to how lichens survive desiccation, and currently nothing is known about the contribution of the associated microbial communities to this process. This is especially relevant, as lichens harbour diverse bacterial communities [19], which are persistent members of the holobiont [20–22]. Essential contributions of bacterial communities to the functioning of the holobiont were revealed by multi-omics technologies, including nutrient supply, resistance against biotic/abiotic stress, support of photosynthesis by provision of vitamin B_12_, fungal and algal growth support by provision of hormones, and detoxification of metabolites [23, 24]. Previous studies have shown that the bacterial colonizers are equipped with various mechanisms to withstand toxic lichen metabolites and survive repeated desiccation and hydration cycles [25]. It was our hypothesis that the microbiota would most likely adapt to the varying environmental conditions with adjustments at gene expression levels and we assumed that these temporal transcriptional variations in the associated microbiota would contribute to the symbiotic processes of the entire system. We therefore studied the activity of bacterial communities associated with the lung lichen *Lobaria pulmonaria* (L.) Hoffm. using a sampling design resembling a natural hydration cycle and analysing the response to contrasting hydration stages using a metatranscriptomic approach.

## Materials and Methods

### Collection of lichen samples

*L. pulmonaria* thalli were sampled in the Austrian Alps (Johnsbach, N 47°32′35″, E 14°37′38″; 1175 m above sea level) from the bark of a mountain maple (*Acer pseudoplatanus* L.). Sampling was conducted in the late morning hours, after a rainy period of 2 weeks on the 28 June 2014. The temperature and relative humidity were measured with a Easylog EL-USB-2 data logger (Lascar Electronics, PA, USA) starting 12 days before the sampling date. The average temperature was 15.9 °C, with a minimum of 8 °C and a maximum of 30 °C (std.: 4.2 °C). The average relative humidity range was between 35.5 and 101.5%, with an average of 80.7% (std.: 16.6%). Overall, 18 lichen thalli were collected from maple bark (area < 1 m²) to compare them at two different physiological stages – *i.e*. hydrated and desiccated thalli, respectively. Therefore, nine thalli were sampled with sterile tweezers, cleaned of macroscopic contaminations (*e.g*. moss, bark, and insects) and immediately transferred into a 15-ml Sarstedt tube with RNAlater stabilization solution (Ambion, Life Technologies, Germany). Less than 2 min elapsed between lichen collection and fixation in RNAlater. Additionally, nine thalli were collected and subsequently dried in the laboratory at room temperature for 4 weeks. This dehydration method was chosen due to forgoing studies, that showed that the lichen microbiome remains stable over longer periods of time [19], and also to survive even decade-long storage when dried out [26]. All of the 18 selected thalli were rather young, as estimated by their thallus size, and visibly healthy.

### Community mRNA extraction

The preparation of a lichen transcriptome dataset with substractive hybridization was described in a recent multi-omics study [25]. Total RNA was extracted using the TRIzol® Plus RNA Purification Kit (Ambion, Life Technologies), according to the manufacturer’s protocol. Briefly, 1 ml TRIzol® Reagent per 50–100 mg of tissue sample was added to each lichen thallus and then homogenized at room temperature using the FastPrep™ Lysing Matrix E and a FastPrep®-24 Instrument (MP Biomedicals, Germany) for 3 × 30 s at 6.0 m/s with 1 min in-between cooling on ice. After a 5-min incubation step on ice, chloroform was added to the homogenized samples and further incubated with subsequent separation of the phenol-chloroform and aqueous phase by an additional centrifugation step at 4 °C. Subsequently, RNA was purified using the spin column-based PureLink® RNA Mini Kit (Ambion, Life Technologies, Germany). The samples were kept on ice in between the processing steps to reduce enzymatic RNA degradation. Following the first cleanup step, the total RNA was further purified with an RNeasy mini kit (Qiagen, Germany) according to the manufacturer’s protocol to eliminate remaining TRIzol traces. The RNA integrity was measured using the Agilent 2100 Bioanalyzer (Agilent Technologies, CA, USA) to assure sufficient quality before further processing.

Subtractive hybridization via sample-specific biotinylated rRNA probes was used to remove both bacterial and eukaryotic ribosomal RNA (according to Stewart et al. [27] and Kukutla et al. [28]). Briefly, the eukaryotic and prokaryotic SSU and LSU rRNA gene fragments were amplified using designed primer sets, based on corresponding metagenomic DNA sequences (see Table S1). Subsequently, the purified PCR products (Wizard SV Gel and PCR Clean-Up System, Promega, Germany) were used as DNA templates for the preparation of biotinylated anti-sense rRNA probes via in vitro transcription, according to the manufacturer’s protocol (MEGAscript® T7 Transcription Kit and MEGAclear™ Transcription Clean-Up Kit, Ambion, Life Technologies, Germany). Finally, the rRNA was subtracted from the total RNA with streptavidin-coated magnetic beads after hybridization with the specific biotinylated anti-sense rRNA probes. Quality-checked mRNA of three separately processed lichen thalli was pooled equimolar. Strand-specific cDNA library preparation of total and depleted RNA and Illumina HiSeq 2500 paired-end sequencing was performed by GATC Biotech AG (Konstanz, Germany).

### Transcriptome assembly and annotation

The raw sequence data consisted of Illumina HiSeq paired-end (2 × 100 bp) RNA-seq reads, sequenced from three replicates samples each in the dry and hydrated conditions, respectively. The number of read pairs per sample ranged from 59,968,245 to 71,991,873. Assembly of the RNA-Seq reads into contigs was performed using the Trinity (v2.1.1; [29]) RNA-Seq *de novo* assembly software, with default parameters. Sequence data from all six samples were assembled together in a combined assembly. A total of 884,943 transcripts consisting of 743,495,150 bases was produced, with a contig N50 value of 2540 and an average contig length of 840 bases. The transcriptome was annotated using the Trinotate (v3.0.0; https://trinotate.github.io/) annotation suite, which analyzes both assembled transcript DNA sequences and predicted protein sequences, combining the results for functional annotation. The coding regions within transcripts were predicted using TransDecoder (v2.0.1; https://transdecoder.github.io.com/TransDecoder), based on homology with Swiss-Prot proteins and Pfam protein domains. A total of 585,453 coding sequences were predicted, coding for the same number of proteins. Both the Trinity-assembled transcript sequences and Transdecoder-predicted protein sequences were searched against the Swiss-Prot databank, using Blastx and Blastp programs, respectively [30]. In addition, a replicate quality check was performed using the Trinity utility tools to ensure that the three replicates of each hydration condition were comparable (Fig. S1).

### Transcript abundance estimation and differential expression analysis

Transcript expression levels were quantified using RSEM (v1.2.28; [30]). The quantification was performed separately for each of the replicate samples, based on the alignment of RNA-Seq reads to the *de novo* assembled transcripts. For differential expression analyses, the RSEM-generated transcript abundance matrix was analysed using the Bioconductor limma/voom [31] package. Transcripts with a minimum fold change (FC) of 2 and a maximum FDR of 0.01 were extracted. Two different sets of transcripts and their expression matrix were generated. The first set included the transcripts upregulated in the dry condition, and the second set included those upregulated in the hydrated condition.

### Taxonomic and functional assignments

Kaiju [32] was used to perform highly sensitive taxonomic assignments, using maximum exact matches of the query sequences translated to amino acids and protein database sequences for assigning taxa to input sequences. The analysis was performed with a database consisting of the NCBI non redundant (NR) protein sequences from all bacteria, archaea, viruses, fungi, and microscopic-sized eukaryotes, respectively. Functional assignments were performed for all differentially expressed transcripts. The bacterial transcript sequences were searched against the NCBI NR database using Blastx, and the results were imported into MEGAN (v6.7.1; [33]). Functional assignments at different SEED [34] levels were compared for bacteria in general and for distinct taxonomic groups therein, respectively.

### Availability of the generated transcriptome datasets

The data are available on MG-RAST [35] under the identifiers mgm4745782.3, mgm4780346.3, mgm4780354.3, mgm4780579.3, mgm4781288.3, and mgm4781296.3. One of the datasets, ‘mgm4745782.3’, was utilized to extract specific sequences that were integrated into a meta-omics study [25].

## Results

**Fig. 1:**
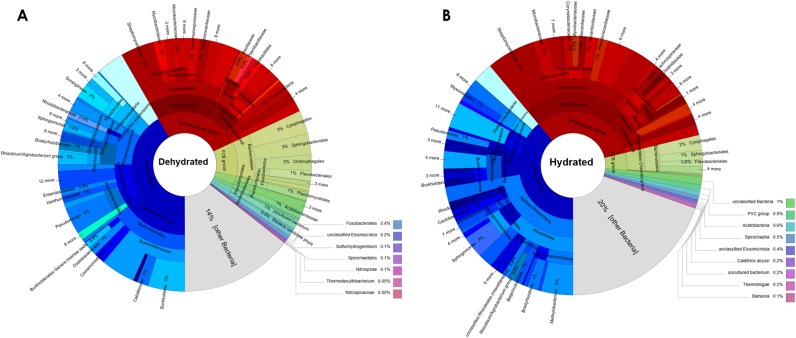
Assignment of upregulated transcripts to bacterial lineages under dehydrated (**a**) and hydrated (**b**) conditions. Bacterial transcripts were separately assessed for upregulation under the two distinctive hydration stages of the lichen host. The two datasets were used to assign corresponding taxa to the filtered transcripts. The output of the analysis was visualized with Krona charts [51]. Segment coloration was adapted to distinguish between higher taxonomic ranks: *Proteobacteria* (blue), *Actinobacteria*, *Firmicutes*, *Cyanobacteria* (red), *Bacteroidetes* (green-yellow)

Transcripts that were upregulated in the hydrated and dehydrated stage of the lichen holobiont were separately assessed. Following transcript assignment to phylogenetic groups, the taxonomic composition was visualized for each of the two hydration stages (Fig. 1). Under both conditions, upregulated transcripts assigned to *Proteobacteria* were predominant, with 41.7% under dry and 38.9% under hydrated conditions, respectively. When phylogenetic assignments within *Proteobacteria* were analysed at a higher resolution for the dry stage, *Alphaprotebacteria* accounted for 9%, *Betaproteobacteria* for 16%, *Gammaprotebacteria* for 9.1%, and the Delta/Epsilon subdivision for 4.1% of all differentially expressed bacterial transcripts. *Rhizobiales* were the most prominent order (4.4% of all differentially expressed bacterial transcripts) within *Alphaproteobacteria*, and *Bradyrhizobiaceae* (1.1%) the most abundant family therein. *Betaproteobacteria* were mainly represented by *Burholderiales* (14.9%; order) and *Burkholderiaceae* (11.1%; family). *Xanthomonadales* (1%; order) were common within the *Gammaproteobacteria*. For the hydrated stage, phylogenetic assignments within the *Proteobacteria* showed predominance of *Alphaproteobacteria*, accounting for 22.3% of the differentially expressed transcripts within the bacteria. *Betaproteobacteria* accounted for 6%, *Gammaprotebacteria* for 5.2% and the Delta/Epsilon Subdivision for 2.9%. *Rhizobiales* (12.7%; order), *Sphingomonadales* (3.8%; order) and *Methylobacteriaceae* (4.4%; family) were identified as the main representatives of the *Alphaproteobacteria*. *Betaproteobacteria* were mainly assigned to the order *Burkholderiales* (4.8%), while *Gammaproteobacteria* were mainly assigned to the *Pseudomonadales* (1.5%). Other phylogenetic assignments for the dry condition showed a subordinate presence of *Actinobacteria* (15.7%), *Firmicutes* (6.6%) and *Bacteroidetes* (10.3%). *Pseudonocardiaceae* (1.3%) and *Microbacteriaceae* (0.8%) were identified as the main families within the *Actinobacteria*. *Firmicutes* included primarily *Lactobacillales* (0.9%; order), *Bacillaceae* (0.9%; family) and also the genus *Paenibacillus* (0.8%). *Chitinophagaceae* (2%; family) were common within the differentially expressed *Bacteroidetes* assignments. The results show that the hydration status of the lichen considerably affects the active fraction of the microbiome.

**Fig. 2:**
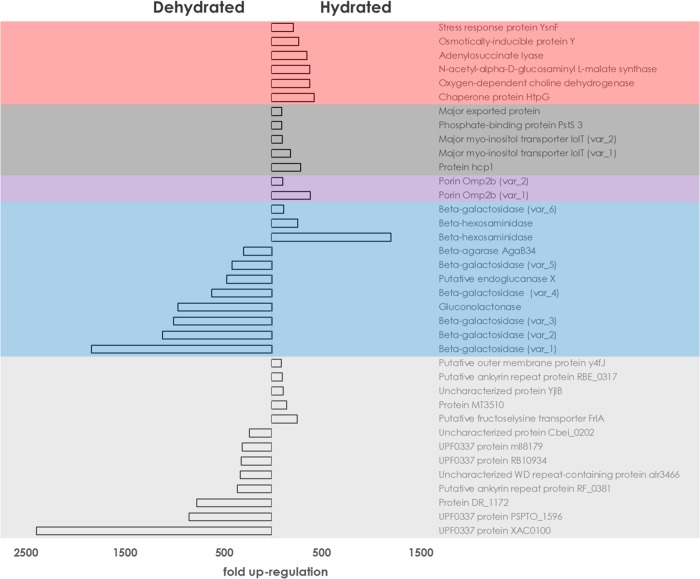
Global analysis of bacterial transcripts with differential expression. Annotated transcripts with the highest-fold change under each hydration stage were analysed to obtain a general overview of bacterial gene expression. Transcripts with a maximum false discovery rate (FDR) of 0.01 were utilized for the underlying analysis. Different background colours indicate distinct functional groups. Red: stress response; Dark grey: Transport systems; Violet: Porins; Blue: Carbohydrate metabolism; Light grey: Uncharacterized proteins

The hydration status of *L. pulmonaria* had extensive implication on the overall transcription activity on the bacterial level (Fig. 2). To obtain a general overview of the impact of dehydrated and hydrated conditions on the transcription activity, the functional assignments of the top 30 genes under each condition, i.e. those with the highest-fold change differences of the expression level were analysed in greater detail (highlighted in Table S2). The fold change of upregulated transcripts ranged between 94 and 2371 when this threshold was applied. Under dry conditions, various variants of transcripts coding for carbohydrate degradation were prevalent. The other group of related transcripts with repeated occurrence was assigned to mechanisms of stress protection. In the carbohydrate degradation group, five distinct β-galactosidase transcripts were prominent, with five distinctly upregulated transcripts under dry conditions. They were all assigned to members of the *Gammaproteobacteria* and the transcript with the highest upregulation showed an almost 2000-fold change (FC: 1816) when compared to the wet condition. In addition, a gluconolactonase (FC: 945), the putative endoglucanase X (FC: 453), and beta-agarase AgaB34 (FC: 281) were found within this group. In the stress protection-related group, the chaperone protein HtpG (FC: 429) showed the highest upregulation, followed by an oxygen-dependent choline dehydrogenase (FC: 383), a N-acetyl-alpha-D-glucosaminyl L-malate synthase (FC: 381), an adenylosuccinate lyase (FC: 355), the osmotically inducible protein Y (FC: 272) and the stress response protein YsnF (FC: 219). The ketone body metabolism included two transcripts in dehydrated lichens, aldo/keto reductase slr0942 (FC:214) and acetoacetate metabolism regulatory protein A toC (FC: 213), respectively. Among the functions that were not assigned to any of the two major groups, a transcript coding for a dimethyl-sulfide monooxygenase was 1511-fold increased in the dehydrated samples. Moreover, eight proteins without annotated function were upregulated in the dry stage of the lichen, showing fold changes between 223- and 2370-fold. Only one transcript coding for an antimicrobial compound, a gene annotated as oleandomycin polyketide synthase, showing a 210-fold change when compared to the hydrated stage, was identified. For the hydrated condition, a higher diversity existed in known functions among the top 30 upregulated transcripts, when compared to the dehydrated condition. The functional group with the largest number of transcripts included various transport proteins. Other transcripts belonging to a common group were assigned to carbohydrate degradation and tRNA modification. Five transcripts that code for either proteins without annotated function or proteins annotated as “putative”, were also identified in hydrated lichens. Their fold changes ranged between 98 and 256. In the large transport-system-associated group, protein hcp1 (FC: 290) accounted for the highest change in the hydrated samples, followed by two variants of the major myo-inositol transporter IolT (FC: 187 and 106), phosphate binding protein PstS 3 (FC: 103) and a major exported protein (FC: 99). For carbohydrate degradation, two variants of β-hexosaminidase (FC: 1199 and 263) and one transcript coding for β-galactosidase (FC: 122) were identified in the hydrated stage. In contrast to the taxonomic assignments for the dehydrated stage, all transcripts related to carbohydrate degradation were associated to the Gram-positive *Firmicutes*. The group related to tRNA modifications included a polyribonucleotide nucleotidyltransferase (FC: 129), tRNA(Glu)-specific nuclease WapA (FC: 122) and methionine-tRNA ligase (FC: 106). Two variants of porin Omp2b assigned to the predominant order of the *Rhizobiales* were upregulated under the hydrated condition (FC: 390 and 112). The transcript with the highest upregulation under this condition was assigned to 2-epi-5-epi-valiolone synthase (FC: 1792), which plays an important role in biosynthetic pathways related to antimicrobial compounds. In summary, both hydration conditions can be correlated with distinct patterns of microbiome function.

**Fig. 3:**
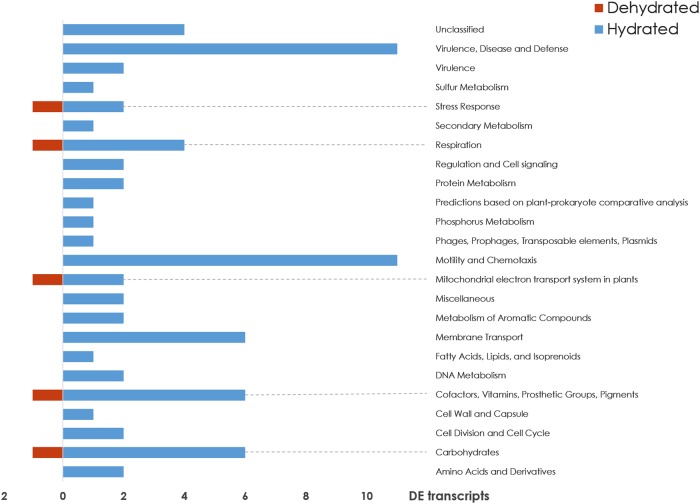
SEED clustering of *Rhizobiales*-associated transcripts. All upregulated transcripts that fall within a distinct SEED category were included in the visualization. The dataset was filtered for taxonomic assignments at order level. All transcripts that were assigned to *Rhizobiales* were subjected to SEED categorization within standard categories

Members of the *Rhizobiales* order were previously identified as central components of the lichen microbiome [36, 37]. The present metatranscriptome analysis shows differing activity of this predominant order under hydrated and desiccated conditions. A total of 75 transcripts, which were upregulated under hydrated conditions and assigned to *Rhizobiales*, were assignable to a SEED function (Fig. 3). In contrast, only five transcripts, which were upregulated under the dry condition within the *Rhizobiales* fraction, were assignable to a SEED category. The SEED categories with most assigned transcripts under hydration were motility and chemotaxis (11 transcripts) and virulence, disease and defense (11 transcripts), respectively. Additional categories included carbohydrates (6 transcripts), cofactors, vitamins, prosthetic groups, pigments (6 transcripts), membrane transport (6 transcripts), respiration (4 transcripts) and four transcripts without classification. In the desiccated stage, only a single upregulated transcript was identified for each of the following categories: carbohydrates, cofactors, vitamins, prosthetic groups, pigments, mitochondrial electron transport system in plants, the respiration and stress response respectively. Previous studies, which addressed the role of microbes in the lichen symbiosis, have identified highly abundant porins on the protein level [24, 38]. Our transcriptome datasets also show high expression levels of porin Omp2b, especially under hydrated conditions (Fig. 3). Therefore, more detailed analyses were performed to determine the variability of these transcripts. All transcripts that were assigned to porin activity in the GO ontology database [39] were filtered for further protein database searches. This led to the identification of a total of 26 transcripts with homology to proteins with porin activity, which were classified in more depth (Table 1). Comparisons within Swiss-Prot (https://www.ebi.ac.uk/uniprot) indicated that the transcripts were 24.4–86.2% identical to porin-related database entries. Transcripts were assigned to the 31 kDa outer-membrane immunogenic protein, outer membrane protein IIIA, Porin Omp2a, Porin Omp2b and putative outer membrane protein y4fJ via comparisons to the Swiss-Prot database.

**Table 1:** *Rhizobiales*-associated porin transcripts in the *L. pulmonaria* metatranscriptome

Transcript ID	GO molecular function	Swiss-Prot Entry	BLASTX against Swiss-Prot (% identity)
TRINITY_DN90730_c0_g1_i1	Porin activity	31 kDa outer-membrane immunogenic protein	34.85%
TRINITY_DN507480_c0_g1_i1	Porin activity	31 kDa outer-membrane immunogenic protein	24.40%
TRINITY_DN176849_c0_g1_i1	Porin activity	31 kDa outer-membrane immunogenic protein	35.14%
TRINITY_DN176196_c0_g1_i1	Porin activity	31 kDa outer-membrane immunogenic protein	41.33%
TRINITY_DN170897_c0_g4_i3	Porin activity	31 kDa outer-membrane immunogenic protein	42.86%
TRINITY_DN170897_c0_g1_i1	Porin activity	31 kDa outer-membrane immunogenic protein	36.36%
TRINITY_DN183927_c3_g8_i8	Porin activity	Outer membrane protein IIIA	86.21%
TRINITY_DN186154_c2_g5_i1	Porin activity	Porin Omp2a	46.75%
TRINITY_DN186154_c2_g9_i1	Porin activity	Porin Omp2b	39.62%
TRINITY_DN183927_c3_g8_i5	Porin activity	Porin Omp2b	31.06%
TRINITY_DN183927_c3_g8_i4	Porin activity	Porin Omp2b	28.95%
TRINITY_DN172633_c0_g1_i1	Porin activity	Porin Omp2b	25.53%
TRINITY_DN162614_c1_g1_i1	Porin activity	Porin Omp2b	41.86%
TRINITY_DN186442_c5_g1_i5	Porin activity	Putative outer membrane protein y4fJ	62.16%
TRINITY_DN186442_c5_g1_i4	Porin activity	Putative outer membrane protein y4fJ	62.16%
TRINITY_DN186442_c5_g1_i2	Porin activity	Putative outer membrane protein y4fJ	62.16%
TRINITY_DN186442_c5_g1_i1	Porin activity	Putative outer membrane protein y4fJ	62.16%
TRINITY_DN186154_c2_g4_i3	Porin activity	Putative outer membrane protein y4fJ	36.54%
TRINITY_DN179502_c1_g1_i5	Porin activity	Putative outer membrane protein y4fJ	33.73%
TRINITY_DN590939_c0_g1_i1	Porin activity	No hit	–
TRINITY_DN56543_c0_g1_i1	Porin activity	No hit	–
TRINITY_DN351126_c0_g1_i1	Porin activity	No hit	–
TRINITY_DN186442_c5_g4_i2	Porin activity	No hit	–
TRINITY_DN186154_c2_g5_i4	Porin activity	No hit	–
TRINITY_DN179206_c0_g1_i1	Porin activity	No hit	–
TRINITY_DN163693_c0_g1_i1	Porin activity	No hit	–

Transcripts with an identical % identity (BLASTX) but different transcript IDs align with the same database hit region that is potentially conserved. These transcripts are distinguishable outside the shared region.

## Discussion

**Fig. 4:**
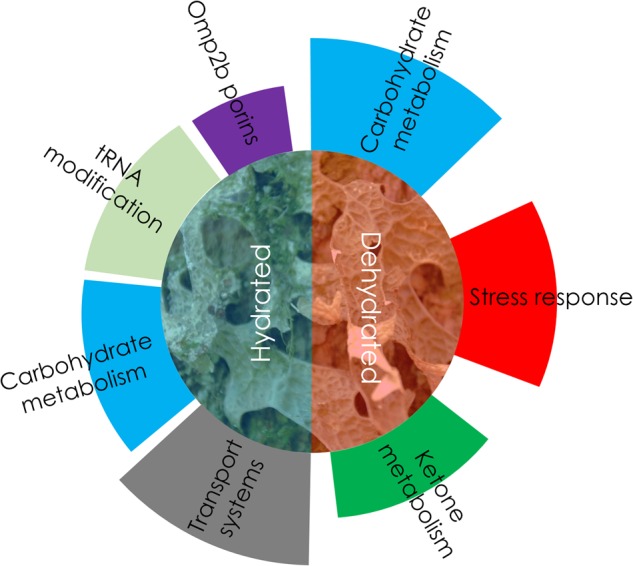
Schematic visualization of the main functional groups activated under hydrated and dehydrated conditions. Both hydration stages were accompanied by distinctive functional activities of the microbiome. Functional groups are highlighted with distinct colours of the outer segments. The size of the segments correlates with the number of up regulated transcripts in the respective group

This study provides the first insight into the microbiome activity for the two distinct stages of hydration in lichens. It is the first study to provide a detailed insight into a microbiota’s response to a condition that is adverse for most known organisms. We have shown that host-associated bacterial communities can be welladapted to dehydration by stress protection and through changes to the overall metabolic activity. Moreover, we were able to show that the dehydrated stage was mainly correlated with activation of stress-reducing mechanisms within the bacterial microbiome, especially showing gene activity related to the lowering of osmotic stress (schematic visualization in Fig. 4). In addition, the degradation of sugar polymers was highly activated during this stage and accompanied by ketone metabolism. The presence of upregulated transcripts associated with specific metabolic pathways could be indicative for a distinct survival strategy of the holobiont. The interconnectivity of the distinct pathways provided by different bacterial lineages remain to be further explored. Interestingly, the combined metabolic profile of the microbiome strikingly resembled cellular processes of higher organisms during fasting. In particular, we were able to show that several taxonomically unrelated bacterial lineages were involved in a functional transition that resembled a ‘fasting metaorganism’. Switching to the ketone body metabolism during food deprivation is a common strategy in multicellular organisms [40]. Together with the activation of other specific enzymes, this indicates a transition to a lipid-based nutrition. Algae and lichens are known to contain lipid bodies for storage, which are highly diverse and unique in structure (contain partly exotic lipids like betaine lipids, n-alkanes or halogenated lipids), but similar in function to those of other eukaryotes [41]. The catabolic turnover of lipid reserves, instead of glucose for providing energy, is well known in humans and animals. The mobilisation and conversion of lipids also creates osmotically active substances to retain water for macromolecular integrity. Interestingly, starvation response and ketogenesis were correlated with an extended life span of animals (e.g. [42]). Whether the similar correlations are also associated with the well known longevity of lichen symbionts is pending for further research. Nevertheless, this is the first time that transcriptional signals of ketogenesis have been associated with the bacterial partners contributing to a symbiosis. Other fasting-induced processes concern the carbohydrate metabolism in various life forms. When glucose becomes scarce at the cellular level, the β-galactosidase repressor is deactivated in addition to other metabolic transitions to facilitate the utilization of additional carbohydrates [43]. The cell wall of the mycobiont (fungal partner of the lichen symbiosis) usually includes complex polysaccharides [44, 45]. We hypothesize that bacteria colonizing the lichen surface can intrude into deeper layers of the gelatinous matrix of the mycelial cortex of *L. pulmonaria*, which is mainly held together by exopolysaccharides from deliquescent outer layers of fungal hyphae. By reaching these deeper layers, terminal saccharides become accessible to the fungus and can be utilized by the β-galactosidase producers, when other nutrients are scarce. Previously, we were already able to observe bacteria entering the cortex of *Lobaria* [40]. Because the bacterial density is substantially lowered beneath this layer, we also assume that bacteria are finally resorbed by the fungal symbiont in deeper layers. In otherwise oligotrophic habitats, this could represent a complementary source of nutrients (phosphorous in particular), which is known as a limiting factor of lichen growth [46]. In this context, lichen-associated bacteria were already shown to be involved in nutrient scavenging and phosphate mobilization [47].

Hydration of the metaorganism resulted in a higher functional diversification and during this stage; the well-described lichen associates *Rhizobiales* showed upregulation of distinct transcripts. Members of this bacterial order were found to be integral parts of the lichen microbiome [41] providing various beneficial functions, which support the symbiosis [40]. In the framework of this study, distinct changes in expression levels were found for the *Rhizobiales*-assigned Omp2b porins in hydrated lichens. Bacterial porins are primarily involved in the passive transport of hydrophilic molecules of various sizes and charges across the membrane. In a different context, bacteria-derived porins have already been shown to play a role in the prevention of apoptosis in yeast cells [48]. In a metaproteomics study with the same lichen model published by Eymann et al. [24], porins were also found to be abundantly present among bacterial proteins. Notably, the samples for that study were collected during the hydrated stage, which corroborates our present results. Porin-dependent mechanisms might directly affect the mycobiont of the metaorganism during hydration processes. When distinct lichen species rehydrate, various oxygen radicals with damaging properties are formed [14, 49]. A mechanism that was primarily linked to pathogenicity [48] might also have a beneficial effect in the lichen symbiosis. In the context of the lichen symbiosis, the porins formed by the highly abundant *Rhizobiales* might prevent cell death in the mycobiont due to interference with fungal apoptosis factors. However, the detailed role of this specific porin group in the lichen holobiont will need to be characterized further.

We have provided evidence for the distinctive role sharing of the predominant bacteria in the lichen metaorganism under contrasting host conditions. Experiments conducted with a representative set of desiccated and hydrated samples indicated hydration-dependent specification of the microbiota, while the previous differentiation of ‘feeders’ and ‘protectors’ among the functional colonizers was also supported [25]. Owing to their sensitivity to changing conditions, the roles of specific and generalist lichen-associated bacteria in the lichen system might vary in the symbiosis, which is in agreement with a metaorganism concept. The data of bacterial transcriptomes still need to be fully integrated in transcriptomics studies of lichen hydration, which has so far focused only on the eukaryotic partners [17]. Moreover, the limitations of the experimental design of this study can be solved by in situ monitoring of specific partners or pathways. For this study, a sufficient period of drying under controlled lab conditions was chosen because hydration conditions in nature were hardly predictable with weather conditions and daily fluctuation of hydrations. The storage under lab conditions also caused a time shift in the sampling. Due to their stability, long life span and slow metabolism [50], this difference seems to be not crucial. The distribution of the main and supporting roles will help to establish a fully integrated picture of the functional course along the timeline of the poikilohydric lifestyle, where all of the organisms involved will be integrated into the symbiotic network reconstruction.

Some of the beneficial mechanisms provided by the microbiota might even be transferable to other systems, including crop plants, which are increasingly subjected to drought due to global warming. The switching to ketogenesis, and fasting-like processes, in microbial symbioses might provide an interesting novel mechanism of symbiotically mediated stress tolerance. Future research needs to work out further details of this hypothesis and the potential for practical applications in a wider range of host–microbe associations.
